# Sequential Evaluation of the National Medical Expenditures for Asthma Care in Japan

**DOI:** 10.2188/jea.14.100

**Published:** 2005-03-18

**Authors:** Shinichi Tanihara, Yasuki Kobayashi

**Affiliations:** 1 Department of Public Health, School of Medicine, Shimane University; 2 Department of Public Health and Occupational Medicine, Graduate School of Medicine, the University of Tokyo.

**Keywords:** asthma, health expenditures, health care costs, morbidity, mortality

## Abstract

BACKGROUND: It has been reported that the prevalence of asthma is on the rise in various countries; but studies on its effects on national medical expenditures are scarce.

METHODS: From the outcome of the “Estimates of National Medical Care Expenditures” and “Patient Survey” compiled by the Statistics and Information Department, Minister’s secretariat, the Ministry of Health, Labour, and Welfare of Japan, medical expenditures on asthmatic patients in Japan were sequentially examined.

RESULTS: It was found that the percentage of asthmatic patients to the general medical care expenditures has been on the steady increase. A closer examination revealed that the percentage of asthmatic outpatients receiving care increased while those receiving care as inpatients decreased.

CONCLUSION: The relationships between the percentage of the number of asthmatic patients utilizing medical services and the expenditures for their care differed between inpatients and outpatients.

Asthma is a chronic disease occurring both children and adults and its crude mortality (3.0 per 100,000 population in 2002) is larger than that of tuberculosis (1.8 per 100,000 population in 2002) in Japan.^1^ It has been reported that the prevalence of asthma^2^^-^^5^ and the number of asthma visits^6^^,^^7^ are on the rise in various countries. The increase in asthma mortality in particular age groups might be influenced by the increased prevalence;^6^ but studies on its effects on national medical expenditures are scarce.^8^

It is believed that multiple factors, such as introduction of new medical technology, an aging population, increases in the disease prevalence or morbidity, and changes in the National Health Insurance System, all contribute to this increase in medical costs. To plan public health policies, it is important to understand the relationship between changes in epidemiologic indices (e.g., prevalence, morbidity, and mortality) for asthma and the medical care expenditures related to its care. In the current study, we investigated the financial effects of asthma on general medical care expenditures in terms of the costs and the number of asthmatic patients in Japan.

## METHODS

From the outcome of the “Estimates of National Medical Care Expenditures” and “Patient Survey” compiled by the Statistics and Information Department, Minister’s secretariat, the Ministry of Health, Labour, and Welfare of Japan, changes in the general medical care expenditure for the care of asthmatic patients, the number of patients, and their proportionate use of medical services were investigated.

For the “Estimates of National Medical Care Expenditures”, the statistics compiled since 1979, in which the 9th Revised International Disease Classification was adopted, were used. The target disease categories were bronchial asthma (according to the Revised 9th International Disease Classification: 493) for the period between 1979 and 1994; and bronchial asthma and status asthmatics (Revised 10th International Disease Classification: J45, J46) for the period between 1995 and 2000.

To study the trends in effects of asthma on general medical care expenditures, annual changes in the cost of asthmatic care costs as a percentage of general medical care expenditures were examined for inpatient and outpatient populations. It was calculated as follows:

$$\frac{\text{the estimated asthma care costs}}{\text{the estimated general medicai care expenditures}}\times 100.$$

For the “Patient Surveys”, the statistics compiled every three years since 1984, in which the current survey method was adopted, were extracted and the estimated number of patients (inpatients and outpatients, as well as the total number of patients) with asthma and their relative utilization of health service were calculated sequentially.

## RESULTS

**Figure 1.**
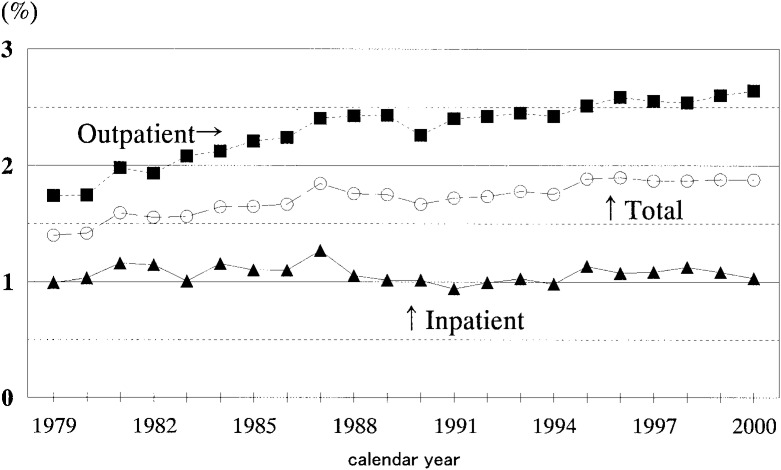
Asthma care costs as a percentage of general medical care expenditures of Japan, 1979-2000. Estimates of National Medical Care Expenditures, reported by the Statistics and Information Department, Minister’s Secretariat, the Ministry of Health, Labour and Welfare of Japan.

The asthma care costs as a proportion of the general medical care expenditures are calculated for inpatients, outpatients, and total patients in 1979 and thereafter (Figure 1). The total expenditure for asthma care for year 2000 was 450 billion yen (117 billion yen for inpatients and 334 billion yen for outpatients). As a proportion of the general medical care expenditures for the same year (23,961 billion yen, i.e., 11,343 billion yen for inpatients and 12,618 billion yen for outpatients), it was 1.88% (1.03% for inpatients and 2.64% for outpatients).

The asthma care costs as a percentage of general medical care expenditures increased by 0.48% between 1979 and 2000; but this increase was not evenly distributed throughout the period. Having increased from 1.40% to 1.85% between 1979 and 1987, it remained around 1.75% between 1988 and 1994 (except 1.67% for 1990). After reaching a high of 1.90% in 1995, it plateaued within a very narrow range of 1.87 and 1.88%.

The proportion of the outpatient asthma care costs to the outpatient general medical care expenditures was greater than it was to total general medical care expenditures for any year. Furthermore, the percentages themselves continued to increase: 1.74% in 1979 rose to 2.64% in 2000.

The proportion of the inpatient asthmatic care to the inpatient general medical care expenditures was lower than it was to total general medical care expenditures, ranging from 0.94 in 1991 to 1.27% in 1987. Although there were some fluctuations in those figures, it generally remained within a 1% variation, showing no obvious increases such as those seen in outpatient care for the corresponding time period.

The estimated percentages of asthmatic patients receiving care and those (i.e., inpatients, outpatients, and the sum of these two) to the total number of patients receiving care per 100,000 population is shown in Table 1 (data taken from “Patient Survey”). The number of asthmatic inpatients per 100,000 population was 17 in 1984, which followed a pattern of simple diminution to 12 in 1999. The number of outpatients showed a totally different trend: 98 in year 1984 per 100,000 population increased to 126 in 1993, since then the number fluctuated around 120. The total number of asthmatic patients was strongly affected by the number of outpatients: 115 for 1984 followed a pattern of simple augmentation to 140 for year 1993, since then it remained around the 130s.

**Table 1.** Chronological change of the prevalence of asthma and its proportion to all patients, 1984-1999, Japan.

	Calendar year*					

	1984	1987	1990	1993	1996	1999
The number of patients with asthma (a) (per 100,000 population)						
Inpatients	17	16	16	14	13	12
Outpatients	98	107	111	126	123	120
Total	115	123	127	140	136	132

The number of all patients (b) (per 100,000 population)						
Inpatients	1118	1174	1214	1146	1176	1170
Outpatients	5285	5426	5554	5589	5824	5396
Total	6403	6600	6768	6735	7000	6566

The proportion of asthma patients to all the patients (a/b, %)						
Inpatients	1.52	1.36	1.32	1.22	1.11	1.03
Outpatients	1.85	1.97	2.00	2.25	2.11	2.22
Total	1.80	1.86	1.88	2.08	1.94	2.01

* : “Patient Survey” is conducted every three years.

The proportion of asthmatic patients seeking care to the total patients also differed between inpatients and outpatients. For inpatients, 1.52% in 1984 followed a simple declining pattern to about two-thirds of the previous figure (1.03% for 1999). For outpatients, the proportion increased from 1.85% (1984) to 2.25% (1993) and fluctuated in a range of 2.1 to 2.3% thereafter. The total number of asthmatic patients requiring care increased, following the pattern for the outpatients and reached a maximum of 2.08% in 1993.

## DISCUSSION

The current study revealed the following three fluctuating patterns in terms of the number of asthmatic patients and the costs incurred by them: (1) The costs of asthmatic care as a proportion of general medical care expenditures increased steadily, mainly resulting from increases in the costs of caring for ambulatory asthmatics; (2) the proportion of asthmatic outpatients seeking medical services to the total number of patients increased, though for inpatients the comparable metric decreased over the years; and (3) In spite of reductions in the percentage of asthmatic inpatients to the total inpatients, the percentage of costs of asthmatic inpatient care to the total inpatient care costs has not shown much change.

The increases in the costs of asthmatic care are concordance with the recent rise in the prevalence of asthma and the increase in the proportion of asthma patients to the total number of patients. The changes in prevalence are expressed by both an increase in self-reported asthma^2^^-^^5^ and an increase in the number of patients who could be cared for on an ambulatory basis due to advancements in therapeutics.^6^^-^^9^ Consequently, the relative expensiveness of inpatient care for asthma may be explained by an increase in the ratio of asthmatic patients who are in a more dolorous condition, requiring more costly treatment.

The statistics used in the current study, namely “Estimates of National Medical Care Expenditures”, were estimated from the sampling studies of the statements of the medical care reimbursement sheets. Thus not included are those patients who have not sought medical attention. Neither was consideration given to the effects of indirect medical costs (e.g., lost work hours due to absenteeism necessitated by asthmatic attacks). When multiple disease entities are listed on the medical care reimbursement sheets, the selection of a major entity depends on the description given there. While diagnostic criteria are not necessarily uniform in the reimbursement system, there are occasions when asthmatic severity may be judged by the number of prescribed drugs.^10^^,^^11^ However, within the framework of the medical insurance system in Japan, the disease entity entered on the medical care reimbursement sheets and drugs prescribed for treatment are stringently examined by the insurer. Therefore the information recorded on the medical reimbursement sheets is supposed to be relevant and reliable to examine medical services to asthmatic patients.

The Health Insurance System of Japan applies to all citizens and it is safe to assume that the results of the current study accurately represent the trends in asthmatic care expenditure and utilization of the care by patients throughout the country. Interestingly, the relationships between the percentage of the number of asthmatic patients utilizing medical services and the expenditures for their care differed between inpatients and outpatients; it is easily predicted that the increasing number of asthmatic outpatients would accelerate the rise of general medical care expenditures. In the future, it will be necessary to evaluate the outcomes of asthmatic patient care, such as mortality and quality of life, considering changes in health care-seeking patterns for patients affected by the introduction of new therapeutic modalities.
